# Rapid Fabrication of Electrophoretic Microfluidic Devices from Polyester, Adhesives and Gold Leaf

**DOI:** 10.3390/mi8010017

**Published:** 2017-01-09

**Authors:** Christopher Birch, Jacquelyn A. DuVall, Delphine Le Roux, Brandon L. Thompson, An-Chi Tsuei, Jingyi Li, Daniel A. Nelson, Daniel L. Mills, James P. Landers, Brian E. Root

**Affiliations:** 1 Department of Chemistry, University of Virginia, Charlottesville, VA 22904, USA; cb2xj@virginia.edu (C.B.); jacquelynduvall@gmail.com (J.A.D.); dsl4b@virginia.edu (D.L.R.); blt8gt@virginia.edu (B.L.T.); at3ae@virginia.edu (A.-C.T.); jl8pr@virginia.edu (J.L.); dan6n@virginia.edu (D.A.N.); 2 TeGrex Technologies, Charlottesville, VA 22910, USA; dlm7b@virginia.edu; 3 Department of Mechanical and Aerospace Engineering, University of Virginia, Charlottesville, VA 22904, USA; 4 Department of Pathology, University of Virginia, Charlottesville, VA 22904, USA; 5 Applied Research Institute, University of Virginia, Charlottesville, VA 22904, USA; ber2a@virginia.edu

**Keywords:** microfluidic, adhesive, centrifugal, DNA

## Abstract

In the last decade, the microfluidic community has witnessed an evolution in fabrication methodologies that deviate from using conventional glass and polymer-based materials. A leading example within this group is the print, cut and laminate (PCL) approach, which entails the laser cutting of microfluidic architecture into ink toner-laden polyester sheets, followed by the lamination of these layers for device assembly. Recent success when applying this method to human genetic fingerprinting has highlighted that it is now ripe for the refinements necessary to render it amenable to mass-manufacture. In this communication, we detail those modifications by identifying and implementing a suitable heat-sensitive adhesive (HSA) material to equip the devices with the durability and resilience required for commercialization and fieldwork. Importantly, this augmentation is achieved without sacrificing any of the characteristics which make the PCL approach attractive for prototyping. Exemplary HSA-devices performed DNA extraction, amplification and separation which, when combined, constitute the complete sequence necessary for human profiling and other DNA-based analyses.

## 1. Introduction

Human DNA profiling processes have been transitioning to a microfluidic format from the macroscale at a growing rate over the last twenty years [1]. The process comprises a concatenation of steps; sample introduction, DNA extraction, polymerase chain reaction and finally electrophoretic separation of the amplified loci, providing the user with a unique profile pertaining to the sample donor. The microfluidic realm offers a platform to integrate those steps, enabling automation and ease of use. In many ways, the microfluidic or ‘lab-on-chip’ device has evolved incrementally since its beginnings, which can be attributed to the regular introduction and surge in popularity of new core materials [2]. Consequently, the evolution of microfluidic DNA analysis devices has largely been shaped by those changing paradigms. The story frequently invoked to summarize this describes an emergence of glass materials in the early 1990s, followed by a gradual but significant shifting of attention to various polymers, such as polydimethylsiloxane (PDMS), polymethylmethacrylate (PMMA) and cyclic olefin copolymer (COC) [3]. The field was later enriched by a steady flow of alternative materials such as paper, hydrogels, wax, ink and other readily-available plastics, which would appear amidst the waves of innovation propelled by those leading materials. Initially, glass possessed the characteristics considered ideal for microfluidic operations, including high transparency, hydrophilicity, solvent compatibility and amenability to functionalization, however, the fabrication process, which employs a combination of photolithography, wet etching and high temperature bonding, took multiple days, slowing throughput, making mass-manufacture difficult and expensive [4]. Furthermore, the photolithography necessitated a custom-designed mask for each new design, slowing prototyping down further. In response, several polymers were proposed to offer advantages over glass, however, no single emerging material was free from drawbacks. Elastomers, such as PDMS, were ideal for rapid prototyping with greater material permeability benefiting those exploring microbiology applications including cell culturing. Unfortunately, fabrication involved casting a liquid in a mold and was not conducive to mass-manufacture demanding further redevelopment, if commercialization were to be achieved. Thermoplastics, such as PMMA and COC were utilized by embossing or injection molding channel architecture with a customized stamp. In contrast to PDMS, this offered greater potential for mass-manufacture, but did not serve as an efficient prototyping platform [4,5]. The lingering limitations associated with these traditional materials provided justification for lesser-established techniques to be brought into the spotlight to have a real impact on the field. A good example of this is the print, cut and laminate (PCL) approach [6], which comprises CO_2_ laser ablation of microfluidic architecture into ink-toner coated polyester sheets and multilayer device formation by lamination of those sheets. This rapid journey from design to device offered a robust and inexpensive platform for prototyping and has recently been enhanced by implementing centrifugal fluidic control. The features combined, have enabled successful application to DNA extraction [7], amplification [8] and separation [9], all of which are essential in human genetic profiling.

It can be argued that no current application necessitates the mass-manufacture of low cost disposable devices more than that of DNA analysis, which is why the marriage of human profiling to the PCL technique holds so much promise. However, while the methodology has inspired this and a multitude of other successful projects [10,11,12,13], there are concerns regarding the strength and long-term stability of the ink-toner as a binding agent, eliciting caution from those hoping to send their various innovations in to the market. What is required is an alternative to the toner, which would prevent layer delamination and render the devices sufficiently stable for their intended applications, but would not convolute the development and/or add expense to the manufacture of the devices themselves. Currently there is a plethora of commercially-available adhesives that can be procured to introduce this greater stability, but many possess undesirable characteristics, which may make handling and device assembly problematic. In other words, it is critical that for an adhesive to be deemed suitable to augment the final device, its properties must not jeopardize the simplicity of the PCL process.

In this communication, we report the exploitation of a heat-sensitive adhesive (HSA) to fabricate microfluidic devices for each of the three key steps in DNA profiling; DNA extraction, polymerase chain reaction (PCR) and electrophoretic separation. The challenges accompanying these processes are many and include chemical inertness of the surface, heat stability and adhesive strength. All operations incorporate centrifugal transportation and mixing of fluids along with necessary heating steps to carry out individual assays. In doing so, we showcase the benefits of fabricating with this material throughout the duration of prototyping and elevate its significance further with additional emphasis on the potential for mass-manufacture of robust and durable devices.

## 2. Materials and Methods

### 2.1. Device Fabrication

Fabrication of the devices was inspired by the PCL methodology [6], in which multilayer microfluidic devices comprise polyethylene terephthalate (PeT) transparency sheets bonded by lamination. The heat-sensitive adhesive (HSA) introduced to this fabrication technique was purchased from Adhesives Research (EL-7970-39, Glen Rock, PA, USA). Lamination was carried out using a common office laminator and all architecture was cut with a CO_2_ laser cutting device. The design concept for the three exemplary devices presented here can be scrutinized in a series of papers published by our laboratory [7,8,9], however the fundamental principles are as follows: DNA extraction device consists of a storage unit for liquid extraction reagents, adjacent to a chamber containing a buccal swab with the device supported by an additional PMMA layer (3 mm) to provide the required volume. The reagents are introduced to the swab centrifugally and the chamber is subsequently heated for 2 min to perform the extraction. The PCR device provides a chamber to accommodate sample and PCR reagents accessed by two channels leading to outlets. Once filled, the PCR chamber is thermally cycled to amplify target sequences which can then be analyzed off-device. Finally, the separation device comprises three chambers containing a separation gel and a chamber containing PCR product. The device also has an injection-molded COC layer attached to provide the separation channel and cross-T. First the separation gel is centrifugally-loaded into the separation domain, followed by centrifugal loading of the sample into one arm of the cross-T. Finally, this device is subjected to voltages to separate the amplified DNA fragments. DNA separations are supported by integrated electrodes, comprising layers of gold leaf, pressure-sensitive adhesive and PeT, also described in greater depth by Thompson et al. [9]. Electropherograms were obtained using a single-color separation system built in-house.

### 2.2. Reagents

Reagents used for DNA extraction comprised 88 μL of distilled water (DI H_2_O), 10 μL 10× prepGEM TM saliva buffer, and 2 μL of prepGEM TM Saliva EA1 enzyme (ZyGEM Corporation Ltd., Hamilton, New Zealand). The heat for DNA extraction was provided by a common laboratory heat plate (VWR^®^ Hotplate/Stirrer, Radnor, PA, USA), 75 °C for 2 min. Resultant DNA concentrations were quantified using both a fluorescence assay (PicoGreen^®^, Invitrogen, part of Life Technologies Corp., Carlsbad, CA, USA) and a NanoDrop 3300 fluorospectrometer (Thermo Scientific, Wilmington, DE, USA) Reagents for PCR comprised a 10 μL custom 10 plex master mix: (Promega, Madison, WI, USA), 7.5 μL custom 10-plex primers (Promega, Madison, WI, USA), 2 μL 2800 M control DNA (2.5 ng/μL) (Promega, Madison, WI, USA) and 5.5 μL H_2_O. Thermal cycling parameters were 96 °C for 1 min, followed by 30 cycles of 94 °C for 15 s, 59 °C for 25 s, and 72 °C for 20 s. A final extension at 60 °C for 2 min then concluded the process. The separation polymer used was a hydrophobically-modified polyacrylamide (1.5%/2.5%) in a tris/borate/EDTA + urea buffer. The sample comprised 1 μL internal lane standard (ILS) (Promega, Madison, WI, USA), 3 μL 6-plex PCR product (Promega, Madison, WI, USA), and 6 μL of HiDi Formamide (ThermoFisher Scientific, Waltham, MA, USA). Before use, the sample was heated at 95 °C for 3 min, followed by snap cooling in ice for 3 min. All conventional capillary electrophoresis was performed on an ABI 310 instrument.

## 3. Results

### 3.1. Fabrication

While the chemical nature of this commercial adhesive material remains proprietary, it is understood that it functions in two key stages. The first stage is a simple softening and hardening of a ‘tack’ induced at temperatures that approach 100 °C, while the second phase is chemical and irreversible, occurring between 120 and 160 °C. Here, both phases are initiated to bind the layers together by passing them through an office laminator, operating at a temperature between 200 and 220 °C. The adhesive could not be handled easily when removed from a protective backing, due to its elasticity and lack of rigidity. For this reason, HSA layers are laid on to both sides of a PeT sheet with the protective backing still attached and then bound by lamination.

**Figure 1:**
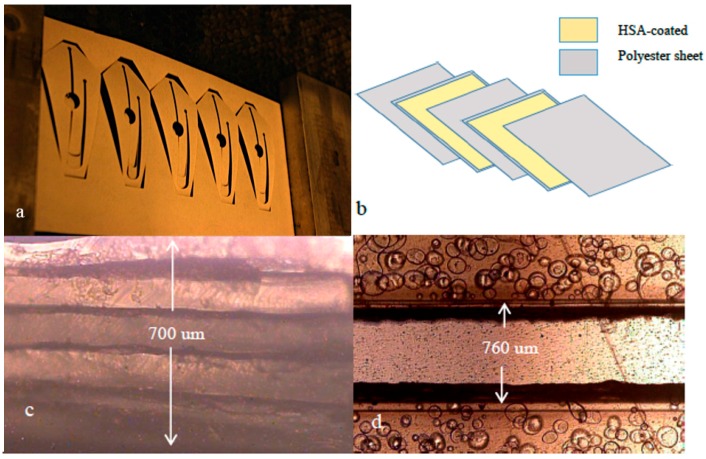
(**a**) Photograph of single layer of polyester transparency with heat-sensitive adhesive (HSA) and backing on each side, post-cut [8]; (**b**) Schematic describing the separate layers comprising the device. PeT (grey) and HSA (beige) layers; (**c**) Microscopic image of device cross section showing five layers; (**d**) Microscopic image of device channel.

Once laminated, channel architecture can be cut in these layers (Figure 1a) and the backing subsequently be removed, leaving the PeT with the adhesive exposed on each side. Finally, each HSA-coated layer can be assembled with accompanying layers (Figure 1b) and again be laminated forming the multilayer device. This sequence of events could be carried out in less than five minutes, comparable to fabricating devices with ink-based toner. This must be emphasized, as incorporating this HSA in to the fabrication process allows the user to retain the ability to assemble the devices with minimal skill and control. This was not the case when attempting fabrication with comparably-priced pressure-sensitive adhesives (not heat-sensitive), which demanded a high level of precision from the user, including those with extensive experience of the PCL process.

**Figure 2:**
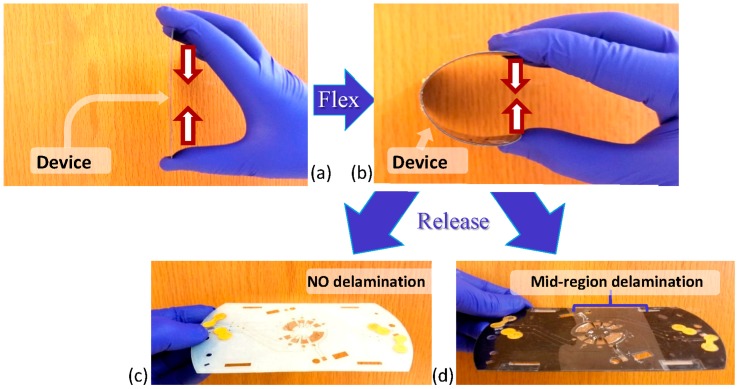
Images of (**a**) Device before flex; (**b**) Device during flex; and (**c**) A device post-flex, which was fabricated using HSA-based binding; and (**d**) A device post-flex, which was fabricated using traditional toner-based binding. A comparison of images (**c**,**d**) demonstrates that the HSA-based devices withstand physical stresses, whereas the toner-based devices delaminate.

The thickness of the multilayer PeT/HSA ranged between 660 and 700 μm across the devices. As the PeT are 100 μm thick and contribute five layers, the thickness of the four layers of HSA post-lamination can be estimated at approximately 40–50 μm each. This cross section is illustrated in Figure 1c. In addition, Figure 1d shows an image of a typical channel (width 760 μm), showing a clean cut with no discernable damage to channel walls. There is, however, some visible roughness along the sidewalls, which is likely due to the softening and hardening of the adhesive in this region. This roughness would affect approximately 6% of the total channel wall. Finally, in order to demonstrate the material strength of the device, a five layer PeT/HSA device (pared disc-shaped) was stressed mechanically by manual bending. Device showed substantial resilience to bending, in fact, only when opposite ends of a device lay parallel (angle of 180°) were there signs of fracturing or delamination (Figure 2). Upon release, the device returned to its original shape. This demonstrates that the devices can tolerate significant manual stress with no permanent damage, and contrasts the stability of the toner-based predecessor, which would experience varying levels of delamination under smaller stresses (<10°) (Figure 2d). With the goal being field deployment of these devices with different applications designed for many different real-world environments, it is critical that a device is not susceptible to damage from handling or transport. While the discs could be stabilized with a layer of a material exhibiting a higher Young’s Modulus, this adds fabrications steps and, hence, cost. It is also acknowledged that additional components or layers will likely have an influence on this flex tolerance, however the flexibility of the PeT/HSA exceeds that of comparable device materials such as PMMA and COC [3], making the devices more resilient to real-world use.

### 3.2. Fluidics and DNA Assays

**Figure 3:**
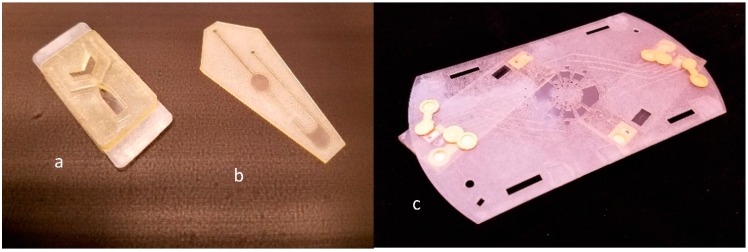
(**a**) DNA extraction [7]; (**b**) PCR device [8]; (**c**) Separation device [9]. Note, DNA extraction device has additional polymethylmethacrylate (PMMA) layer (3 mm) to increase volume for swab and reagents. Separation device has attached cyclic olefin copolymer (COC) component to provide separation dimensions.

The devices presented here, specifically designed for DNA extraction, amplification and electrophoresis (Figure 3), were developed to support human DNA profiling and, as such, present three demonstrative assays that all pose unique challenges to the materials used. The ‘DNA extraction device’ (Figure 3a) requires storage of 100 μL of extraction reagents followed by transport of this fluid (driven by centrifugal force at 1000 RPM for 30 s) to an adjacent chamber containing a swab. Following this, the swab chamber is subjected to 75 °C for 2 min. The device withstood the high temperature and centrifugally-induced fluidic pressure with no breach of the materials. DNA extractions yielded concentrations of 3.3 ± 1 ng/μL (*n* = 10) from buccal swabs, which were shown to be amenable to subsequent PCR amplification.

For the ‘PCR devices’ (Figure 3b), while this process does not require any fluid transport, the materials challenge here is to show robustness when subjected to extensive temperature cycling, spanning the range of 23–95 °C. This also was achieved without the device being compromised (*n* = 10), and all fluid was retrieved for analysis upon completion. Analysis of this amplified product using conventional capillary electrophoresis demonstrated that PCR on the HSA devices was successful, as deemed by the appearance of full profiles exhibiting peaks all over 2000 RFUs. Each sample peak describes the length in base-pairs of an individual genetic marker, with the group of 10 peaks collectively characterizing the unique genetic makeup of the sample donor. The size of each marker can be determined by using the incorporated size ladder as reference.

**Figure 4:**
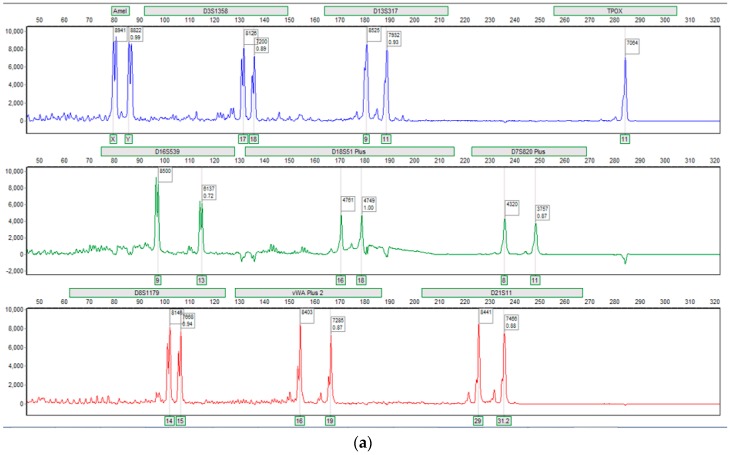

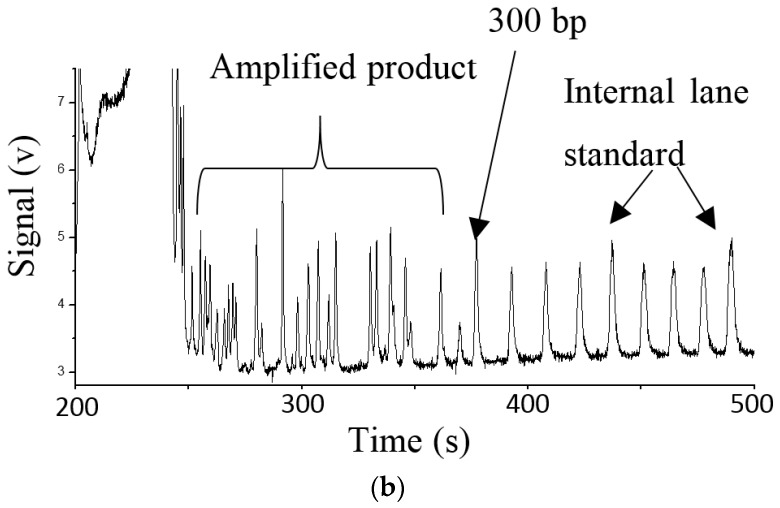
(**a**) DNA profile obtained from off-chip capillary electrophoresis separation of 10 loci amplified on HSA-based PCR device [8]. Electropherogram provided is a screen shot reproduced from Genemarker^®^ software (SoftGenetics, State College, PA, USA). PCR consisted of 30 cycles and took 19 min. *y*-axis range from 0 to 10,000 RFUs; (**b**) Electropherogram obtained when separating 6 loci on-chip on single-color separation system built in-house [9].

The ability to successfully amplify directly on a HSA-based device, as well as from DNA extracted from cells on a separate HSA-based device, demonstrated that the materials can accommodate PCR without inhibition. Furthermore, the materials did not require any surface treatment prior to use to eliminate surface-absorption of PCR components. This contrasts several popular materials, such as glass, which requires a surface coating to reduce or eliminate component adsorption. A typical profile can be seen in Figure 4a.

The ‘electrophoresis device’ presents several materials challenges not seen in either the extraction or PCR device. The first is that a polymer, used as a ‘sieving matrix’ or ‘gel network’ during DNA electrophoresis [9], must be transported from a storage chamber to a separation domain, filling the electrophoresis architecture in a bubble-free manner so that electrical connectivity is comprehensive. Since the separation channel has a cross-sectional area of only 80 μm × 80 μm, there is an inherent increased pressure induced by spinning at 1600 RPM for several minutes. The device was able to tolerate the transportation of the gel at these speeds with no observed delamination or damage to the device. Following this, voltages ranging from 200 to −1800 V were applied to this domain in order to electrophoretically separate the DNA fragments. The device supported this process with no observed issues, and the DNA fragments were separated successfully, with the profile not looking dissimilar to that obtained in a glass device [14]. This result also demonstrated the compatibility of the HSA material with the gold leaf electrodes, which had thus far only been integrated into PeT/ink toner devices. Using this combination of PeT, HSA and gold leaf, the three devices supported processing of a buccal swab to allow for the generation of a human genetic profile (Figure 4b).

It is clear that future applications may necessitate higher temperatures, higher spin speeds and/or higher fluid flow rates, driven by assays that necessitate different architecture and exposure of fluids to different temperatures. The latter, in turn, has a corresponding influence on thermal transfer. Targeted processing timescales may necessitate faster flow rates or fluidic mechanisms such as mixing chambers or siphon valves and, hence, may also necessitate higher spin speeds. To date, the devices have tolerated all necessary heating steps (up to 105 °C) and have withstood centrifugal speeds of up to 3000 RPM with no observed delamination or breach. All devices described can be manufactured for a materials costs of less than $1 USD, with fabrication timescales being limited only by the laser-cutting step (maximum 30 min for devices described). Due to this, it is foreseen that rate of manufacture could undergo significant optimization and scale up in the near future.

## 4. Conclusions

The devices showcased here possess a set of properties which equip them for use in an environment laden with challenging scenarios and conditions. The ability to have manufactured devices of this nature in such a rapid and facile fashion makes it an attractive choice for DNA analysis applications and it is expected that a fully integrated human profiling tool will soon stand on this platform. Moreover, the true robustness of these devices makes the technique applicable to a multitude of applications ranging from environmental and agricultural to detection of drugs and explosives, all of which often benefit from equipment that can be taken beyond the laboratory and allow for high-volume one-time use and disposability at little expense.
